# Occurrence of influenza antivirals and resistance development in influenza A viruses in aquatic environments: A risk assessment

**DOI:** 10.1371/journal.pone.0358447

**Published:** 2026-09-21

**Authors:** Hanna Söderström Lindström, Sara H. Norström, Chaojun Tang, Richard H. Lindberg, Josef D. Järhult

**Affiliations:** 1 Department of Epidemiology and Global health, Umeå University, Umeå, Sweden; 2 Department of Natural Science, Design and Sustainable Development, Mid Sweden University, Östersund, Sweden; 3 Department of Chemistry, Umeå University, Umeå, Sweden; 4 Department of Medical Sciences, Uppsala University, Uppsala, Sweden; National Taiwan Ocean University, TAIWAN

## Abstract

Influenza antivirals (IAs) have been detected in aquatic environments inhabited by dabbling ducks, the natural reservoir of influenza A virus (IAV), raising concerns about the development of antiviral resistance. Because novel human IAV strains often contain genetic material of avian origin, this may contribute to resistance in viruses with pandemic potential. This study aimed to assess the environmental risk posed by four IAs—oseltamivir carboxylate (OC), zanamivir (ZA), peramivir (PE), and amantadine (AM)—based on their potential for environmental release, environmental stability, and induction of antiviral resistance. The assessment combined data from new experiments on (1) environmental release and (2) environmental stability of PE, AM, OC, and ZA, with results from previously published in vivo experiments in a mallard model examining (3) resistance development to OC, PE, and ZA in IAV. The risk of environmental release was assessed as high for OC, AM, and PE, and very high for ZA. Environmental stability ranged from very high to low, in the order PE > AM > OC > ZA. The potential to induce resistance in IAV was similar for PE and OC, and lower for ZA. Overall, the environmental risk ranking was PE > OC > ZA, with PE and OC posing the highest risks. Prudent use of IAs requires balancing the risk of resistance development against clinical benefit. In cases of complicated influenza or in high-risk patient groups, the clinical benefits are substantial and justify IA use. However, in uncomplicated influenza among otherwise healthy individuals, the clinical benefit is limited, and the risk of resistance development should be carefully considered. Among the evaluated antivirals, ZA showed the lowest environmental risk and should be preferred when feasible.

## Introduction

Influenza A virus (IAV) causes recurrent seasonal epidemics and occasional pandemics. Antiviral drugs, collectively referred to as influenza antivirals (IAs), are widely used for treatment and prophylaxis. Adamantanes, such as amantadine (AM), were previously common but their use has declined due to side effects and widespread resistance [1]. Over recent decades, neuraminidase inhibitors (NAIs) have become the preferred class of IAs. Oseltamivir (Tamiflu) is the most widely used; due to poor bioavailability, it is administered as the prodrug oseltamivir phosphate, which is rapidly converted to the active metabolite oseltamivir carboxylate (OC) by endogenous esterases. Two additional NAIs are in clinical use, zanamivir (ZA) and peramivir (PE). More recently, a new class of IAs—polymerase inhibitors—has been introduced, with baloxavir currently in clinical use.

Resistance development to NAIs has been well documented both *in vitro* and *in vivo* [2]. The global spread of an OC-resistant seasonal IAV strain 2007−8 (the dominant circulating A/H1N1 strain of that season carried the substitution H274Y) demonstrated that resistance can occur with maintained viral fitness in a suitable genetic background [3]. Waterfowl – mainly dabbling ducks – constitute the natural IAV reservoir [4]. IAV pandemics occur by introduction of new strains to humans. These new strains have often evolved in intermediate hosts, but the genetic material originates from IAVs circulating in waterfowl. Thus, the genetic makeup of new human IAVs depends on the genetic landscape of waterfowl IAVs.

If IAs enter aquatic environments through wastewater discharge, they may exert selective pressure for resistance in IAV infecting dabbling ducks. Accumulation of resistance-associated mutations in this reservoir could contribute to the emergence of drug-resistant pandemic strains.

Development and production of vaccines against a new IAV strain is a slow process; therefore, large global stockpiles of oseltamivir have been established as part of pandemic preparedness [5]. These stockpiles would be useless in case of an OC-resistant IAV pandemic, underscoring the importance of understanding environmental exposure to IAs and their potential to drive resistance in natural reservoirs.

The environmental risk of IAs has been known for more than 15 years. OC was the first of the IAs to be detected in the aquatic environment [6] and has since been reported in river water in concentrations up to 865 ng/L [7,8]. As with most pharmaceuticals, its occurrence is primarily due to wastewater discharge. Other IAs have subsequently been detected in rivers and wastewater, including ZA [8,9], PE [9], and AM [9,10], indicating that conventional wastewater treatment plants (WWTPs) have limited removal efficiency for these compounds. In contrast, advanced treatment methods such as ozonation have been shown to achieve high removal efficiencies [9,11]. For example, Azuma et al. reported >90–99.9% removal of OC, ZA, and PE following ozonation after biological treatment [12]. Similarly, we have demonstrated that ozonation of effluents from two conventional WWTPs in Sweden effectively removed ZA, OC, and AM, as well as their major transformation products, with removal efficiency in the order ZA > OC > AM [11].

The environmental stability of IAs influences both the selection and persistence of resistance-related substitutions in IAV. Key removal processes of IAs in the environment include photodegradation [13,14], biodegradation [15,16] and sorption to organic matter [17,18]. Previous studies have shown moderate photodegradation of ZA [19] and low photodegradation of OC [20] in surface water. Azuma et al. investigated the fate of OC, PE, ZA, and AM in 30-day batch experiments and observed minimal biodegradation and negligible sorption to river sediment, indicating that these processes are unlikely to significantly reduce environmental concentrations [9].

Because these four IAs are rarely used simultaneously at concentrations detectable in aquatic environments, controlled batch experiments provide an effective approach for studying their environmental behavior in parallel. Such experiments, using water from different stages of a conventional WWTP, have previously been applied to assess the fate of OC [21] and personal care products [22]. In WWTPs, most active pharmaceutical ingredients (APIs) are reduced to less than 50% due to biological degradation, while removal due to sorption is generally negligible as most APIs have high solubility, low hydrophobicity, and are often negatively charged at neutral pH (and therefore low sorption affinity to biological sludge) [23].

The influenza antivirals included in the chemical batch experiments, OC, PE, ZA and AM were selected based on their clinical relevance for influenza treatment and prophylaxis at the time of the study design. AM was included in the chemical batch experiments because it belongs to a distinct antiviral class (adamantanes) with physicochemical properties and mechanisms of action that differ from NAIs, thereby enabling a broader assessment of antiviral behavior. In addition, AM has also been detected in aquatic environments and was therefore considered environmentally relevant. However, due to the subsequent decline in AM use driven by resistance and adverse effects, it was not included in the viral experiments or the final risk assessment.

The aim of this study was to assess the environmental risk of OC, ZA, PE and AM, based on their risk for occurrence, stability and resistance development in aquatic environments. This assessment integrates data from new chemical batch experiments with results from previously published viral experiments. The chemical batch experiments were designed to evaluate occurrence and stability in parallel, which is difficult to achieve in field studies due to variable usage patterns and low environmental concentrations. Specifically, we examined removal during conventional wastewater treatment, including biological and chemical processes, and environmental degradation through photolysis, sorption to sediment, and biodegradation.

Resistance development was investigated in previously published *in vivo* experiments using a mallard model, in which birds were infected with IAV and exposed to antivirals via their sole water source, simulating natural aquatic exposure conditions.

## Materials and methods

### Chemical batch experiments

Chemical batch experiments were conducted to investigate (1) the removal efficiency of target compounds in water from a conventional wastewater treatment plant (WWTP) and (2) their environmental stability in river water–sediment systems. To distinguish changes resulting from removal, transformation, and degradation processes in wastewater and river water from analytical variability, concentrations measured in time-resolved samples were normalized to the concentrations measured at time zero. This normalization minimizes the influence of both systematic analytical bias and random measurement fluctuations, allowing observed concentration changes to be attributed primarily to removal, transformation, and degradation processes.

### Removal efficiency in water from conventional WWTP

Wastewater grab samples from raw wastewater, chemical treatment, biological treatment, and second chemical treatment, respectively, were collected at Vakin WWTP, Umeå November 2016. At this time, Vakin WWTP used the following treatment steps; chemical phosphorus removal through iron or aluminum salt precipitation, biological treatment using an activated sludge process, and a second chemical phosphorus removal through iron or aluminum salt precipitation, respectively. Each wastewater sample was collected in plastic bottles in triplicate. A mixture of the four IAs OC, PE, ZA, and AM, was added at a concentration of 10 ng/mL of each, to 200 mL of each type of wastewater in 500 mL open plastic beakers under gently stirring. Oseltamivir carboxylate (OC), (RO0640802–002; lot: 01007B243804) and Oseltamivir carboxylate labelled deuterium (OCD3), (RO0604802–004; lot: 511-001-2197/4) was obtained from Roche (F. Hoffmann-La Roche Ltd, Basel, Switzerland). ZA and ^13^C–^15^N_2_ -labeled ZA were kindly donated by GlaxoSmithKline (Stevenage, Hertfordsshire, UK). PE was obtained from Chemleader Biomedical Co. (Shanghai, China).

The batch experiments started within one hour after the wastewater was collected at Vakin WWTP, Umeå November 2016, and was performed at room temperature. The duration time of each batch experiment was following the hydraulic retention time in the WWTP as follows: (i) 3 h in raw sewage water, (ii) 3 h in water from traditional chemical treatment, (iii) 3.5 h in water from biological treatment and (iv) 3 h in water from second chemical treatment, respectively. After the addition of the IA mixture, the compounds interact with the wastewater components in the same way and over the same hydraulic retention time as in the Vakin WWTP. Therefore, this experimental design provides a close representation of the processes occurring in the WWTP. In addition, a batch experiment in Ultrapure water was performed in triplicate to assess possible losses during the experiment and analysis. When the hydraulic retention time was reached, triplicate 10 mL samples were collected. Each sample was directly filtered through a 0.45 µm MFTM-membrane syringe filter and 5 ng of each internal standard (IS) was added. The peramivir-D3 (PE-D3), oseltamivir carboxylate-D8 (OC-D8) and ^13^C-^15^N_2_-zanamivir (ZA-^13^C-^15^N_2_) were used as internal standard for PE, OC and AM, and ZA, respectively.

### Environmental stability

In this study, “batch experiments” refer to closed-tube laboratory incubations designed to quantify the removal of target IA substances from spiked river water–sediment mixtures under controlled, simulated-sunlight irradiation, relative to non-irradiated dark controls held at the same temperature. Each batch unit consisted of a single glass culture tube containing a fixed sediment-to-water ratio (2.1 g dry sediment: 30 mL river water), to which a defined IA substance mixture was added at the start of the experiment (t = 0). Tubes were not resampled; each timepoint represents a separate, destructively sampled set of triplicate tubes that were removed from the light or dark treatment, processed immediately, and discarded after extraction.

River water and sediment samples were collected using plastic containers from the Ume River (63.824501, 20.228483) and the Selånger River (62.405700, 17.224054) in September 2020. Sediment samples were air-dried in a fume hood, sieved through a 2 mm mesh, and coarse debris was removed. Water samples were filtered through 0.45 µm PVDF syringe filters (Fisherbrand) and stored at 4 °C until use. Subsamples were collected for chemical characterization (Table S1 in S1 File).

A total of 66 experimental tubes were prepared, comprising 54 tubes spiked with the IA mixture of OC, ZA, PE, and AM to a concentration of 1000 ng L^−1^ for each compound, representing environmentally relevant concentrations [24] and 12 unspiked blank tubes. For each river system, triplicate spiked tubes were sampled at 0, 18, 46, 96, 215, and 455 h under irradiation, and at 0, 46, 96, and 455 h under dark-control conditions (Table 1). The 0 h samples represented a common baseline and were therefore shared between treatments. Six blank tubes per river system were included, of which three were analyzed immediately to determine background concentrations and three were incubated and analyzed after 455 h (Table 1).

**Table 1:** Overview of the sampling design used for river water–sediment samples originating from the Ume River (Ume) and Selånger river (Sel). Treatments included irradiated and dark control with or without added influenza antivirals (IAs). Numbers indicate the replicate samples collected at each sampling time point. * Denote pre-treatment sampling (t = 0) where three samples from each river was analyzed.

	River water + sediment + IAs				River water + sediment	
	Irradiated		Dark control		Irradiated	
tid (h)	Ume	Sel	Ume	Sel	Ume	Sel
0	3* + 3*				3	3
18	3	3				
36	3	3	3	3		
72	3	3	3	3		
206	3	3				
486	3	3	3	3	3	3
						
	Tot	66				

All samples were irradiated beneath four UV lamps (CLEO Performance 40W-R SLV; 300–400 nm, total irradiance ≈ 22 W m^−2^ or 2.2 mW cm^−2^), corresponding to typical Swedish sunlight conditions. The sample tubes were continuously agitated on a shaking table and the temperature within the irradiation cabinet was monitored daily using a calibrated thermometer and maintained at 20 ± 1 °C through manual adjustment of fan cooling and cabinet ventilation. Dark controls were wrapped in three layers of aluminum foil and placed beneath the UV lamps to ensure exposure to the same temperature conditions as the irradiated samples. Triplicate sample tubes were destructively sampled after 0, 18, 46, 96, 215, and 455 h of exposure, whereas dark controls were sampled after 0, 46, 96, and 455 h (Table 1). To facilitate interpretation, exposure times were categorized into <1 day (18 h), 1–4 days (46–96 h), 5–9 days (215 h), and 10–19 days (455 h). Substances showing no significant removal after 455 h were classified as having removal times >19 days.

The sediment samples were treated according to a previously published method by Golovko [25]. In short, the sediments were air dried under a fume hood. 100 µL of internal standard (IS) was added to the dry sediment which was then extracted with 4 ml of acetonitrile 50% (v/v) 0,1% formic acid and put in an ultrasonic bath (FinnSonicM4, FinnSonic OY, Lahti, Finland) for 15 min. The procedure was then repeated with 4 mL of 30% acetonitrile and 30% isopropanol 0,1% formic acid.

The organic content in the sediment was measured by drying the samples at 105^◦^C until constant weight and then heated at 550 °C for at least 1 h. This duration was sufficient to ensure complete combustion of organic matter, and longer heating times did not affect the measured loss on ignition (LOI) (SS-EN 15935:2021).

Statistical analyses were performed using one-tailed Student’s t-tests, as the hypothesis tests were directed toward decreases in concentration as an environmentally relevant and biologically plausible outcome of the exposure experiments, with a significance threshold of P < 0.05 to evaluate decreases in analyte concentrations relative to the initial (0 h) concentrations. Because the comparisons were pre-specified, no formal correction for multiple testing was applied. For irradiated samples, statistically significant decreases were evaluated with reference to the corresponding dark controls to distinguish treatment-related losses from potential analytical variability or non-photolytic processes. The same analytical procedures and statistical approach were applied consistently across all treatments and sampling times.

### Chemical analysis

The samples were analyzed for IAs by on-line solid phase extraction (SPE) liquid chromatography tandem mass spectrometry (LC-MS/MS). For the analysis of OC, PE and AM the on-line SPE/LC-MS/MS method used has previously been described in detail by Lindberg et al., 2014 [26]. For this study, this method was further developed (Table S2 in S1 File, Electronic supporting information (ESI)) and adding the MS/MS methods for the analysis of PE, OC and AM (Table S3 in S1 File, ESI). For analysis of ZA, the on-line SPE/LC-MS/MS method previously described in detail by Lindberg et al., 2015 was used [19]. Briefly, both SPE/LC-MS/MS methods started with an injection of 1 mL of 10 mL pre-filtered sample. Since the method validation (see below) showed matrix effects that the ISs could not compensate for, matrix matched calibration curves were used for quantification of the PE, OC, AM and ZA levels to avoid this problem. Four-point (range 0.5 to 10 ng/mL) matrix matched calibration curves were made in raw wastewater (influent) to calculate levels in raw wastewater and water after biological treatment and chemical treatment, respectively and in water from second-chemical treatment (effluent) to calculate the levels in second-chemical treatment.

The method development needed for analysis of PE, OC and AM was validated in raw wastewater (influent) and wastewater from the second chemical treatment (effluent) collected in Vakin WWTP for the WWTP batch experiments and compared with Ultrapure water. The method was validated using four-point calibration curves at 0.5, 2, 6 and 10 ng/mL, respectively, and 5 ng deuterated OC-D8 (IS for OC and AM) and deuterated PE-D3 (IS for PE) as IS, respectively. The matrix effects (signal suppression or enhancement) were validated by comparing the slope of the calibration curve of PE, OC, and AM in influent and effluent water with the slope in the Ultrapure water. The total relative recovery was evaluated by comparing the slope of calibration curves of antivirals/IS in influent and effluent water, respectively, with Ultrapure water. Filtration recovery test was conducted in Ultrapure water at a concentration of 2 ng/mL (n = 3) by comparing the signal response of antivirals when added pre and post filtration.

Instrument precision (variations of the instrument response) was validated by three repeated injections of one Ultrapure water, influent and effluent water sample, respectively, including 2 ng/mL of OC, PE and AM. Intra-day precision was validated by consecutive three injections of three Ultrapure water, influent and effluent water samples, respectively, spiked at 0.5 ng/mL and at 6 ng/mL of each compound to study the reproducibility of the analytical method.

### Environmental risk assessment

The total environmental risk of each IAs was assessed based on new data from chemical experiments on environmental occurrence and environmental stability, in combination with data from previously published experiments on resistance development and persistence of resistance in IAV virus. The environmental risk assessment was based on the relative risk between the four IAs, and not on absolute environmental risk.

#### Chemical experiments.

The results of the experiment on removal in conventional WWT, hence environmental release, were used as one of two parameters to assess each IAs risk for environmental occurrence (Table 2). The second parameter was the results of the experiments on environmental stability (Table 3). These risk assessment tables were based on empirical studies and general knowledge on wastewater treatment and biodegradation.

**Table 2:** Assessment of risk for environmental release based on remaining amount after four batch experiments using raw wastewater, chemical treatment, biological treatment, and secondary chemical treatment, respectively.

Remaining amount (%)	Risk level	Colour
<10	very low	
<30	low	
30-50	medium	
50-70	high	
>70	very high	

**Table 3:** Assessment of risk for environmental stability based on remaining amount after exposure for UV-light.

Time with UV-exposure and significant removal*	Assessed risk level	Colour
≤ 1 day	very low	
1 - 4 days	low	
5-9 days	medium	
10-19 days	high	
> 19 days**	very high	

*T-test (P < 0,05), **last sampling point was 19 days (455h).

Generally, removal efficiencies of pharmaceuticals in WWTP are highly compound-specific and can vary from highly removable (80% – 99%), to moderately removable (30% – 70%) and persistent (0% – 30%) [27,28]. In our batch experiments on removal efficiency in water from conventional WWTP, each individual treatment step was studied and hence, the risks will be slightly underestimated based on our chemical experiments. Therefore, we have used a slightly lower percentage (%) for highly removable/ very low to low risk etc. We have also used more steps in the risk assessment to improve the resolution of the risk assessment.

Similar as for removal efficiency in WWTP, biodegradation of pharmaceuticals are highly compound-specific and highly variable. In REACH registration a compound is considered persistent if the half-life in fresh or estuarine water is higher than 40 days [29]. However, we have used 24 h UV-exposure in our batch experiments on environmental stability and hence, the used UV-exposure will increase the biodegradation in comparison to environmental conditions. There we have used a 50–75% increased risk level in our assessment.

#### IAV virus experiments.

Data from previously published experiments in a mallard duck model were used. There are no predefined cut-offs in guidelines or in the literature to categorize such data into different risk levels. Drug exposure needed to induce resistance-related substitutions in IAV (Table 4) was categorized as very high at ≤1 µg/L as IA levels of this magnitude have already been detected in aquatic environments. IA levels of 1–10 µg/L in aquatic environments are likely to occur under certain occasions, e.g., during pandemics and/or near outlets from WWTPs, and thus this risk was categorized as high. Higher IA levels in aquatic environments are increasingly unlikely to occur and the risk was therefore stepwise categorized lower and lower using ten-fold concentration steps. Decreased drug sensitivity induced by resistance-related substitutions (Table 5) was categorized as very low at <4-fold as this is unlikely to change the clinical outcome of treatment. 4–20-fold decreased sensitivity was categorized as low as this could affect treatment and then the risk was categorized higher and higher in 5-fold steps until >500 which was categorized as very high as clinical effect of treatment is very unlikely in this case. Data on persistence of resistance-related substitutions (Table 6) were categorized stepwise from no resistance detected (very low risk as this indicates quick reversion to wild-type and thus highly reduced fitness of the resistant variant) to no indication of reversion to wild-type in multiple subtypes (very high risk as this indicates that resistant variants of several distinct IAVs maintain fitness).

**Table 4:** Assessment of risk for resistance development based on the lowest drug exposure needed to induce resistance-related substitutions in influenza A virus.

Lowest level of drug exposure to induce resistance	Risk level	Colour
>1 mg/L	very low	
100 µg/L-1 mg/L	low	
10 µg/L-100 µg/L	medium	
1 µg/L-10 µg/L	high	
≤1 µg/L	very high	

**Table 5:** Assessment of risk for resistance development based on decreased drug sensitivity induced by resistance-related substitutions.

Decrease in drug sensitivity	Risk level	Colour
< 4-fold	very low	
4-20-fold	low	
20-100-fold	medium	
100-500-fold	high	
> 500-fold	very high	

**Table 6:** Assessment of risk for persistence of resistance.

Persistence of induced resistance	Risk level	Colour
No resistance detected in persistence experiments	very low	
Rapid reversion to wild-type	low	
Some indication of reversion to wild-type	medium	
No indication of reversion to wild-type	high	
No indication of reversion to wild-type (demonstrated for multiple IAV subtypes)	very high	

#### Overall environmental risk level.

The results of the three risk assessments—environmental release (Table 2), stability (Table 3), and resistance development in influenza A viruses (IAV) (Tables 4–6)—were used to assess the overall environmental risk of the three influenza antivirals (IAs): OC, PE, and ZA. No studies on resistance development in IAV have been performed for AM; therefore, AM was not included in the overall environmental risk assessment.

Among the three risk factors, environmental occurrence was considered a prerequisite for environmental risk. However, if the chemical batch experiment indicated a high or very high-risk level for an IA, environmental occurrence was considered to have less influence on the overall environmental risk. Of the two remaining risk factors, resistance development was considered to have the greatest impact on the overall environmental risk level.

## Results and discussion

### Validation of the chemical analysis of AM, OC and PE

Since the method for the chemical analysis of PE, OC and AM was developed for these experiments the methods linearity, injection precision and intra-day-precision were validated, and matrix effects studied.

The linearity of all three antivirals PE, OC and AM was high in Ultrapure water, surface water, and influent water 0.996 to 0.999 and ranged between 0.835 to 0.8857 for effluent water. The injection precision for all water matrices was high (Relative standard deviation (RSD) <13%) (Table S4 in S1 File). The intra-day-precision of the analysis method, in all water matrices, was acceptable (RSD ranged between <13% and<26%) (Table S5 in S1 File).

The signal was generally suppressed in surface and influent water (from 4% to 40%) except for PE in surface water which showed no matrix effect. High ion suppression (from 43% to 60%) was shown for all three IA in the effluent water. The total relative recovery of antivirals in the influent matrix ranged from 101% to 116% which shows that the IS could compensate for the matrix effect except for AM (D3-OC IS) in influent with a total relative recovery of 134%. However, the total relative recovery in the effluent matrix was > 187% showing that the ISs used could not compensate for the matrix effects (Table S6 in S1 File). Therefore, matrix-matched calibration curves were used in the quantification of the PE, OC and AM levels in the influent and effluent batch samples, respectively (Table S7 in S1 File). In addition, due to problems with the IS of ZA during the quantification of the batch experiment samples, matrix-matched calibration curves were used in the analysis of ZA levels (Table S7 in S1 File).

### Batch experiments on removal efficiency in water from conventional WWTP

The variation in OC, PE, AM concentration in triplicate samples and injection, respectively, was low in all four batch experiments (raw wastewater, chemical treatment, biological treatment, and second chemical treatment). Relative standard deviation (RSD) for OC, PE, and AM was in the range 2−5%, 2−9%, and 3−10%, respectively. The variation in ZA concentration in triplicate samples and injections, respectively, was acceptable (7−22%) in all four batch experiments. Compared to average levels (n = 3) in Ultrapure water, the average (n = 3) remaining amounts (%) of PE, OC, AM and ZA in the raw wastewater and after the three treatment steps, respectively, were in the range 54–83% (Table 7). This shows that the four IAs studied are not removed in conventional WWTP and can via treated wastewater be discharged to aquatic environments. Low removal efficiency by conventional WWT has been shown previously for OC [21], and OC, PE, ZA, AM [9], and Baloxavir [12]. The low removal in treatment steps from conventional WWTPs is caused by the high water solubility of the studied IAs (log P −7,1–0,4) and pharmaceuticals in general, which cause low sedimentation in the treatment steps. The total remaining amount summarized for all four batch experiments, was in the following order ZA > OC > PE > AM. This is the same order as highest to lowest water solubility of the four IAs, according to their log P (Table 7). The remaining amounts of OC, PE and AM, were lowest after chemical treatment (55−57%) and followed by second chemical treatment (61−67%). Biological treatment showed low efficiency for all four IAs with about 70% or more remaining amounts (Table 7). Thus, the first chemical treatment showed the highest efficiency of the treatment steps evaluated. For ZA, which has much higher water solubility (log P −7,1, see Table 7) than the other IAs, the remaining amounts (72–83%) were high, hence the treatment efficiency was low, in all four batch experiments.

**Table 7:** Remaining average amounts (%) of influenza antivirals (IA) in wastewater (ww) batch experiments.

IA	Raw ww	Chemical treated ww	Biological treated ww	2nd chemical treated ww	Log P*
OC	67	57	80	65	−2,1
ZA	74	75	72	83	−7,1
PE	59	54	72	67	−1,4
AM	60	55	69	61	0,4

*Log P given by [9].

### Batch experiments on environmental stability

Analysis of sediment extracts from all experimental treatments showed that none of the investigated influenza antivirals (IAs) were detected in the sediment phase at quantifiable concentrations. This indicates that adsorption to sediment did not contribute to the removal of the target compounds during the experiment and that the observed changes in aqueous concentrations were instead attributable to processes occurring in the water phase. Furthermore, none of the target IAs were detected in the blank samples analyzed at either the beginning or the end of the experiment. This demonstrates the absence of measurable background contamination in the river water, sediment, laboratory procedures, or analytical workflow and confirms that all detected concentrations originated from the experimental additions of the IA mixture. As no measurable adsorption to sediment was observed, the environmental removal of the four IAs was attributed primarily to photolytic and biological processes and was generally low at all sampling points (18–455 h). Low removal between sampling points increases the impact of analytical variation on the data. Therefore, we were unable to demonstrate a clear correlation between exposure time and the amount removed in the samples. Consequently, half-lives for the four IAs could not be calculated in this study, indicating that future batch experiments should extend beyond 455 h.

**Fig 1:**
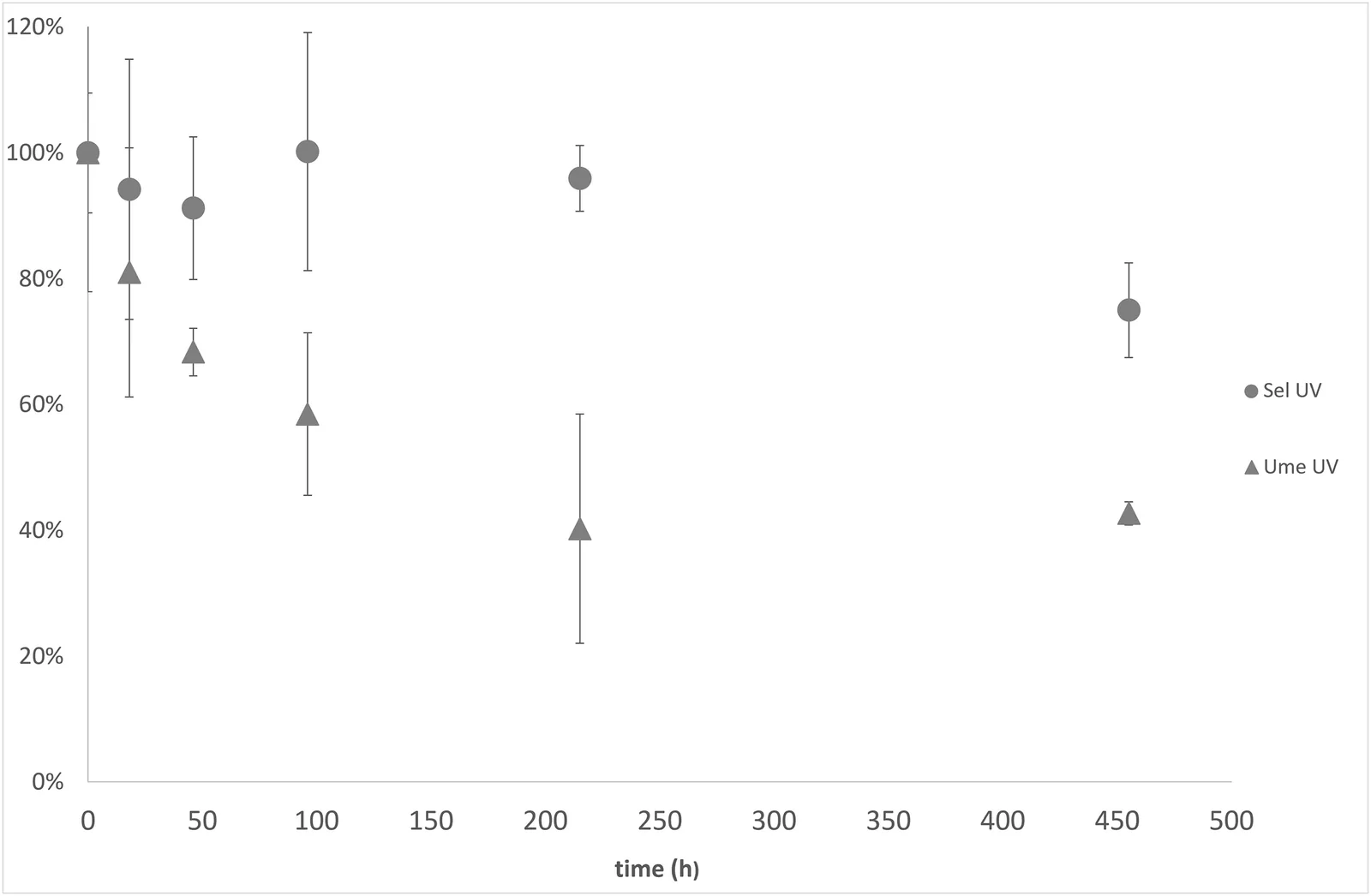
Remaining amount (%) of zanamivir in a river water–sediment mixture exposed to UV light. Data from UV-exposed samples from the Selånger River (Sel) and the Ume River (Ume) were normalized to their respective dark controls. Error bars represent the standard deviation (%).

The only statistically significant amount removed was found for ZA in the Ume River samples exposed to UV-light after two days (50 h) and onward (Fig 1 and Table S8 in S1 File), and a significant decrease could also be seen for OC and ZA in the last sampling point in the Selånger river (Fig 1 and Table S8 in S1 File). ZA, as previously mentioned, has the highest water solubility of the four IAs studied (see log P, Table 7). The amount removed of PE and AM was not statistically significant in any of the sampling points. These results indicate a correlation between increased water solubility and decreased environmental stability. This indicates that different chemical properties of the IAs studied resulted in differences in their removal processes in the environment. This could be one explanation for the differences in amount removed of the IAs studied in the two river matrices (Table S9 in S1 File), which had slightly different pH and sediment composition (Table S1 in S1 File). Thus, we found low removal of the IAs studied after long-term (19 days) of UV irradiation exposure. We have previously shown low removal of OC in surface water after long-term (28 days) UV irradiation with similar experimental conditions (temperature, UV intensity and UV wavelength and OC concentration (1 µg/L) [20]. Azuma et al. (2017 and 2024) [9, 12[also found low removal of ZA, OC, PE, AM and baloxavir in two short-term (7 h) outdoor experiments with similar UV intensity and UV wavelength but 100 times higher concentrations, as in our study. The highest river water concentration of IAs that has been detected is 865 ng/L [7], which is comparable to the IA concentrations used in our experiments on environmental stability.

Due to different experimental conditions (exposure time and IA concentrations) a more detailed comparison was not possible.

### Previously published experiments on resistance development in the natural influenza A virus reservoir

We have previously performed and published several studies on development and persistence of IAV resistance in the natural IAV reservoir, the Mallard duck concerning OC, ZA, and PE [24,30–33]. Although the results of these experiments are already published, we here use the findings to create the risk assessment which has not been performed or published before. In short, resistance development experiments were performed in a room with a single water source, a 170L (1 m^2^) pool, in which desired concentrations of IA were obtained by addition of drug to the water. At the start of the experiment, two Mallards were infected with IAV through oesophageal inoculation. Two new influenza-naive Mallards were then added to the experiment room every third day, thus allowing the viral population to evolve in a situation mimicking nature by repeated replication and transmission to a new host, under selective pressure of IA in the water environment. Persistence experiments were performed similarly but with decreasing levels of/no drug in the water source of the Mallards. Daily fecal sampling of Mallards yielded IAV isolates and sequences to assess resistance genotype and phenotype. Several experiments on resistance development in IAV have been performed on IAs separately, but not as a mixture. This experimental design was chosen to minimize the number of experiments due to financial prioritizations and animal ethics. However, studying the IAs individually could underestimate the risk, as several IAs can occur simultaneously in natural environments. Below we summarize the experimental design and the results.

We have demonstrated resistance development of an H1N1 IAV when infected Mallards were exposed to 0.95 µg/L of OC in their water source, levels similar to what has been detected in river water [24]. When this strain of IAV had acquired IAV resistance through the well-known resistance substitution H274Y, resistance persisted although OC was removed from the water of the Mallards in further animal experiments [32,34,35] This demonstrates that a randomly chosen avian H1N1 IAV can develop resistance when the natural host is exposed to OC at levels similar to what has been found in nature, and that it has a permissive genetic background to harbor such resistance without a detectable fitness loss, as seen by persistence in multiple experiments with no selective pressure. We also demonstrated resistance development in an H6N2 IAV exposed to OC, although at a higher concentration of 12 µg/L [33], and the resistance did not persist when selective pressure was removed [32]. Furthermore, a resistance-related substitution was detected in an H7N9 IAV when exposed to 2.5 µg/L of OC [32].

Experiments exposing Mallards infected with H1N1 IAV (the same strain as used in OC experiments) to ZA demonstrated lower resistance potential as compared to OC based on: i) a lower decrease in viral drug sensitivity as compared to OC (2–17-fold); ii) higher ZA water concentrations needed to induce resistance (at least 10 μg/L); iii) lack of ZA resistance persistence without drug pressure; and iv) multiple resistance-related substitutions were seen during ZA exposure suggesting lack of one straight-forward evolutionary path to resistance [35]. A lower resistance potential as compared to OC was also demonstrated when exposing an H4N2 IAV to ZA as i) 10 µg/L in the water of Mallards resulted in a resistance-related substitution only decreasing drug sensitivity modestly (5.5-fold); ii) 100 µg/L of ZA was needed to detect a substitution substantially decreasing drug sensitivity; and iii) none of the substitutions persisted when drug pressure was removed [36].

For PE, exposing Mallards with H1N1 IAV resulted in rapid acquisition of H274Y at 1 µg/L of PE. No resistance development for PE could be observed in an H4N2 IAV as 1 µg/L of PE inhibited viral replication and no resistance development was seen at 100 ng/L. Infecting Mallards with the resistant H274Y-carrying IAV without exposure to PE showed total persistence of H274Y with no signs of wild-type [34].

### Environmental risk assessment

Environmental occurrence. The relative risk for environmental occurrence of the four IAs OC, PE, ZA and AM were assessed based on the risk of environmental release and stability (Table 8 and 9). The risk for environmental release of the four IAs was assessed to be high to very high. ZA was assessed to have the highest relative risk for environmental release (Table 8). The risk for environmental stability was assessed to be low to very high, and the relative risk for environmental stability was in the following order PE > AM > OC > ZA (Table 9).

**Table 8:** Assessment of the risk for environmental release of the four influenza antivirals oseltamivir carboxylate (OC), peramivir (PE), zanamivir (ZA) and amantadine (AM) based on batch experiments using four wastewater treatments.

	Remaining amount (%) raw wastewater	Remaining amount (%) chemical treatment	Remaining amount (%) biological treatment	Remaining amount (%) second chemical treatment	Assessed risk level
ZA	**very high**	**very high**	**very high**	**very high**	**very high**
OC	**high**	**high**	**very high**	**high**	**high**
AM	**high**	**high**	**high**	**high**	**high**
PE	**high**	**high**	**very high**	**high**	**high**

**Table 9:** Assessment of the risk for environmental stability of the four influenza antivirals oseltamivir carboxylate (OC), peramivir (PE), zanamivir (ZA) and amantadine (AM) based on statistically significant removal in batch experiments with UV-light.

	Remaining amount in UV-exposed samples		Assessed risk level
	Selånger river	Umeå River	
ZA	**high**	**low**	**medium**
OC	**high**	**high**	**high**
AM	**high**	**very high**	**high to very high**
PE	**very high**	**very high**	**very high**

The relative risk for resistance in IAV in Mallards induced by the IAs OC, ZA and PE was assessed based on the results of previous *in vivo* Mallard experiments (Table 10). The level of drug in the water of Mallards needed to induce resistance (Table 4), the highest decrease in drug sensitivity induced (Table 5) and the persistence of resistance when IAV transmitted and replicated under decreasing drug pressure (Table 6) was assessed. The relative risk for inducing resistance in IAV was assessed to be high and similar for PE and OC, and lower for ZA.

**Table 10:** Assessment of the risk for resistance in influenza A virus in the natural reservoir in the environment.

	Level of drug needed to induce resistance	Decrease in drug sensitivity	Persistence to resistance	Assessed risk level
ZA	**medium**	**low**	**low**	**low to medium**
OC	**very high**	**high**	**high**	**high to very high**
AM	**not analyzed**	**not analyzed**	**not analyzed**	**not analyzed**
PE	**very high**	**high**	**high**	**high to very high**

ZA was assessed to have the lowest overall environmental risk of the three IAs due to the lowest relative risk for environmental stability and resistance development, respectively. Based on the highest environmental stability of PE, and comparable risk as OC for resistance development in IAV, PE was assessed to have the highest environmental risk of the three IAs. However, the assessed overall environmental risk of PE and OC were found to be similar. Thus, the environmental risk of the three IAs was assessed to be in the following order PE > OC > ZA (Table 11).

**Table 11:** Assessment of the environmental risk of the four influenza antivirals oseltamivir carboxylate (OC), peramivir (PE), zanamivir (ZA) and amantadine (AM).

	Risk for environmental occurrence	Risk for environmental stability	Risk for resistance development in influenza A virus	Overall environmental risk
ZA	**very high**	**medium**	**low**	**low to medium**
OC	**high**	**high**	**high**	**high**
AM	**high**	**high to very high**	**not analyzed**	**not analyzed**
PE	**high**	**very high**	**high**	**high**

## Conclusions

This is the first study to jointly assess the environmental risk of influenza antivirals (IAs), and the public health risk caused by environmental resistance development in influenza A viruses (IAVs). This comprehensive approach provides new knowledge to support sustainable antiviral use, helping to prevent resistance development in future pandemics and preserve the effectiveness of antiviral stockpiles. Our study shows that the environmental occurrence of IAs, and resistance development in IAVs in aquatic environments, is a concern for all three IAs (OC, PE, and ZA) studied with a high environmental risk of both PE and OC. Our risk assessment was based on virus experiments on individual IAs which could underestimate the risk for environmental resistance development due to cocktail effects in natural environments.

As antiviral drugs constitute a cornerstone in pandemic preparedness, especially in the first phase before vaccines can be mass-produced, it is crucial to work to retain their effectiveness. Given our assessment of the high environmental risk of both PE and OC, and the risk for the resistance developed to be part of a novel IAV with pandemic potential in humans, non-pandemic use should be prudent. This includes balancing benefits of pre-pandemic use with risks of a resistant pandemic IAV. IAs are important in treating complicated diseases and diseases in risk groups and should be used in these cases. However, in uncomplicated influenza in non-risk groups we argue that the clinical benefit is minimal, and if the use is extensive, it will drive environmental occurrence and risk for resistance development. Therefore, we argue that the risk for resistance development should be considered when treating uncomplicated influenza in non-risk groups.

From our risk assessment, it seems beneficial to use ZA rather than oseltamivir phosphate (the pro-drug for OC) when practically possible. PE is the IA with the highest environmental risk in our assessment, hence highest public health risk and other alternatives should be used when possible. As PE is at present only available as an intravenous formulation, it is likely that the use of this drug will not be extensive.

New influenza antivirals such as baloxavir are a very important addition to the treatment arsenal. They should be considered as additions to stockpiles in pandemic preparedness to have access to alternate antivirals with a different mode of action. This will increase the chances of a viable treatment option in case of a resistant pandemic IAV. At the same time, it is crucial to evaluate the risk for environmental resistance development potential of new antivirals to guide prudent pre-pandemic use.

## Supporting information

S1 File Additional supporting tables (Tables S1–S9). (DOCX)
