# Supplementary material for: Occurrence of influenza antivirals and resistance development in influenza A viruses in aquatic environments: A risk assessment
Source: PLoS One. 2026 Sep 21;21(9):e0358447. doi: 10.1371/journal.pone.0358447 (PMC13592582; doi:10.1371/journal.pone.0358447)
Supplement: Supplementary file 1 [file pone.0358447.s001.docx]

**Supporting information**

Table S1. pH of river water and organic content of river sediment.

Table S2. Chromatographic gradient for the Accela LC (A) and Surveyor LC (B) pumps for analysis of Peramivir, Amantadine and Oseltamivir Carboxylate.

| Time, min | Mobile phase composition | | Flow rate, μL/min |
| --- | --- | --- | --- |
|  | water (0.1 % FA) | ACN (0.1 % FA) |  |
| A 0.00 | 95 | 5 | 250 |
| 2.00 | 95 | 5 | 250 |
| 7.00 | 60 | 40 | 350 |
| 8.00 | 0 | 100 | 400 |
| 10.00 | 0 | 100 | 400 |
| 10.01 | 95 | 5 | 250 |
| 12.00 | 95 | 5 | 250 |
| B 0.00 | 100 | 0 | 50 |
| 0.01 | 100 | 0 | 1200 |
| 2.00 | 100 | 0 | 1700 |
| 2.01 | 0 | 100 | 100 |
| 7.00 | 0 | 100 | 1000 |
| 7.01 | 100 | 0 | 1000 |
| 11.00 | 100 | 0 | 1000 |
| 11.01 | 100 | 0 | 50 |

Table S3. Compound Mode Quantification transition, (CE) Confirmation transition, and Tube Lens voltage (V) of Peramivir, Amantadine and Oseltamivir Carboxylate.

| Compound | Mode | Quantification  transition (Collision energy in eV (CE)) | Confirmation  transition | Tube Lens voltage (V) |
| --- | --- | --- | --- | --- |
| PE | + | 329→ 270.2 (20) | 329→ 100.3 (31) | 99 |
| PE_D3 | + | 332→ 273.2 (19) | 332→ 101.3 (32) | 101 |
| AM | + | 152.1→ 135.2 (18) | 152.1→ 79.3 (33) | 72 |
| OC | + | 285→ 138.1 (20) | 285→ 94.3 (29) | 100 |
| OC-D8 | + | 288.1→ 139.1 (19) | 288.1→ 95.2 (30) | 100 |

Table S4. Injection precision for the different matrixes (ultrapure water (MQ), surface water (SW), influent and effluent) of Peramivir, Amantadine and Oseltamivir Carboxylate.

Table S5. Intra-day precision for the different matrixes (ultrapure water (MQ), surface water (SW), influent and effluent) of Peramivir, Amantadine and Oseltamivir Carboxylate.

Table S6. Signal area at different concentrations in ultrapure water (MQ) and raw and treated wastewater for Peramivir, Amantadine and Oseltamivir Carboxylate. Matrix effects (ME) and total relative recovery (TRR) in raw and treated wastewater compared to MQ.

Table S7. Signal area and calculated slope used in the external calibration for Peramivir, Amantadine, Oseltamivir Carboxylate and Zanamivir.

Table S8. Average concentrations of Peramivir, Amantadine, Oseltamivir Carboxylate and Zanamivir (ng/L, n = 3), standard deviations, and P-values from one-sided t-tests comparing each time point to the initial concentration (0 h) within each treatment (irradiated and dark control) in river water from the Selånger River and the Ume River. Significant P-values are shown in bold; bold values indicate a significant decrease in irradiated samples that are not observed in the corresponding dark control. The value with a dotted frame is a significant decrease in a sample with no time matched dark control.

Table S9. Amount (%) of remaining Peramivir, Amantadine, Oseltamivir Carboxylate and Zanamivir in river water after 455 h exposure to UV-light.
